# Integration of Healthcare in Belgium: Insufficient, but There Is Hope: Comment on "Integration or Fragmentation of Health Care? Examining Policies and Politics in a Belgian Case Study"

**DOI:** 10.34172/ijhpm.2022.7179

**Published:** 2022-07-09

**Authors:** Jan De Lepeleire

**Affiliations:** Department of Public Health and Primary Care, Katholieke Universiteit Leuven, Leuven, Belgium.

**Keywords:** Integrated Healthcare Systems, Chronic Care, Fragmentation, Belgium

## Abstract

The maturity of integrated care in Belgium is rather low. The reasons are the country’s complex organization, a lack of leadership and finances, an abundance of pilot projects, very long implementation and change processes, a healthcare system driven by providers and different cultures of action. However, new projects and ongoing research can help overcome these barriers. The primary care zones in Flanders, the National Hospital Plan and the Federal Plan to support mental health in particular are luxating opportunities. Well planned research is urgently needed to confirm the hope these projects arouse.

 IJHPM (*International Journal of Health Policy and Management*) recently published this paper.^1^ To aid understanding, it is important to outline my background and, as a result, any possible conflicts of interest on my behalf or at least biased opinion. I am an academic family physician in Flanders, Belgium. Both in my clinical work and in my research and teaching, I have been heavily involved in the evolution of home care and the organization of healthcare with a special focus on elderly care (long-term care facilities), mental health and palliative care. I am a member of one of the medical syndicates mentioned in the paper and was heavily involved in the preparation of the first and second primary care conference that resulted in the development of the primary care zones – the third case study. Since it was founded in 2020, I have actually been the president in my region of the primary care zone in the south-east of Antwerp.

 Integration or fragmentation of healthcare? This is a very important and relevant question, although this topic is difficult to research. Integrated health services are defined by the World Health Organization (WHO) as “health services that are managed and delivered so that people receive a continuum of health promotion, disease prevention, diagnosis, treatment, disease-management, rehabilitation and palliatieve care services, coordinated across the different levels and sites of care within an beyond the health sector, and according to their needs throughout the life course.”^2^ This case study of three projects in Belgium manages to give an insight into ongoing processes of reform that aim to implement better integration into the Belgian healthcare system. Although such an exercise is difficult in any country, it is almost impossible to implement this in a complicated country like Belgium. The authors manage, however, to explain our system in a deliberate way (Supplementary file 1). The triangulation process they used, combining (grey) literature review and interviews with purposively selected stakeholders and, on top of that, using a semi-quantitative scoring system, is original and effective.

 The paper concludes “that the partial decentralization of healthcare in 2014 has created fragmentation of decisive power which undermines efforts to integrated care.” Data collection ended in September 2019. Six months later, the Coronavirus disease 2019 (COVID-19) pandemic arrived and important gaps and barriers came to the surface: a lack of leadership, a lack of finances, a healthcare system driven by the providers and the different cultures of action.

 An important factor is the lack of leadership, which is an issue that is also addressed in other research.^3^ The paper demonstrates that the retirement of the originator of one of the three projects resulted in a lack of direction. During COVID-19 times, nine ministers (yes nine) where involved in managing the crisis – a very dramatic illustration of fragmentation. This has important consequences. Every minister is competent but no one individual is or feels responsible. As a result citizens neither organizations, health and welfare institutions understand to whom they can raise a problem. Another problem is that real decisions are not taken: the abundance of pilot projects is indeed an important barrier. Some of these projects last more than 20 years and they are neither stopped, embedded in a structural way, or even often without a proper or scientific evaluation. Meanwhile politicians, for the (short) time they are responsible, feel that something is happening and that they did their job.

 This way, a culture of willingness to change is hampered. Even more important is that it takes a (very) long time before changes are decided and implemented. In 2005, a scientific consortium decided to use the Interrai suite of instruments to evaluate the need for care for elderly people in home and residential care.^4^ The first implementation will be in the summer of 2023, 18 years later.

 Besides the lack of leadership, another important barrier is clear: the lack of finances. The COVID-19 crisis requires the governments to implement changes, but the first messages and plans are not reassuring as to the financial opportunities.

 Third, Belgium is marked – not only by a complex institutional design, but also by a culture of concertation and compromises. In all areas of society, a representation of the ‘basis’ is organized. It is noticed as a key component of the system. In healthcare historically, the medical syndicates – since 1870 already – have power in policy making at federal level. This empowers the provider-driven orientation of the system and hampers a more patient-centred system. The concept of the healthcare system became very ‘medical’ oriented and the development of performant public health structures is very weak. This combination becomes an important barrier for change.

 Other barriers are the different cultures between primary and secondary care, healthcare and the welfare sector, like in extenso described by Glouberman.^5,6^

 Despite these problems, there are also positive elements.

- The results of the research under scope, show that there are nuances. Although not quantified, the medical syndicates for instance, do not all have the same ideas and expectations in the three projects as is the case with the sick funds. This means that an open constructive concertation can maybe help with the implementation of projects towards integrated care.
- Furthermore the ongoing reform in Flanders, the third case, creating structural and colloborative platforms, is marked by the researchers as innovative.
- The recent experiences, since 2020, in these primary care zones are positive. What we did learn is that the primary care zones officially started on July 1, 2020 – four months after the first major COVID-19 lockdown. However, most of them started earlier due to a transition phase. At once the public and the government saw the these primary care zones can play and played a role at public, local health level.^7^ This concertation, cooperation and coordination of the medical and welfare settings and institutions, the local responsible persons, the nurses and representatives of the patients and sick funds were able to organize COVID-19 testing, local contact tracing, societal support and the successful COVID-19 vaccination program.

The Federal Government instructed the Federal Knowledge Centre for Health Care to start the “Study 2021-54 (HSR) Integrated care” (https://www.kce.fgov.be) in order to examine the current maturity of integrated care in Belgium, with the aim of formulating recommendations for future policy on integrated care by the summer of 2022. The first results are not yet available but one can expect that the recommendations will stimulate support for change since there is room for improvement.

 Finally we suggest two interesting case studies for further research:

- The ‘Federal hospital plan’ launched in February 2022 and aiming to fundamentally change the organization and renumeration systems of the Belgian hospitals.
- A second case can be in primary care, where the mental healthcare is undergoing an important an in-depth change.

This paper is important and relevant for several reasons. The authors succeeded to untangle very complex and difficult projects in different stages. With an ingenious methodology, that can be an example for other research teams as they revealed the depth of these complex processes. Although it became apparent how big the impact of politics is in this field, the arguments and conclusions are scientific. The result have to be studied by every partner who has responsibility in the reform of Belgian healthcare. Mutatis mutandis, it is also the case for other countries.

## Ethical issues

 Not applicable.

## Competing interests

 Author declares that he has no competing interests.

## Author’s contribution

 JDL is the single author of the paper.
